# Editorial: Plant pest and disease model forecasting: enhancing precise and data-driven agricultural practices

**DOI:** 10.3389/fpls.2026.1924539

**Published:** 2026-08-24

**Authors:** Nathaniel K. Newlands, Vittorio Rossi, Thomas Thomidis

**Affiliations:** 1 Summerland Research and Development Centre, Science and Technology, Agriculture and Agri-Food Canada, Summerland, BC, Canada; 2 Department of Sustainable Crop Production (DI.PRO.VES.), Università Cattolica del Sacro Cuore, Piacenza, Italy; 3 Department of Nutritional Sciences and Dietetics, International Hellenic University, Thessaloniki, Greece

**Keywords:** forecasting, integrated pest management, modeling, monitoring, plant disease, remote sensing

## Introduction

Disease and pest forecasting in plants is a valuable tool in modern agriculture. It helps in the efficient and sustainable management of plant diseases, reduces economic losses, and minimizes the environmental impact of disease control measures. It also underscores the importance of a multidisciplinary approach to agricultural management, integrating science, technology, and effective communication. Predictive models have the potential to revolutionize the management of plant diseases in many ways. These encompass precision in spray applications, reduced costs of spray applications, environmental sustainability, improved disease management, data-driven decision-making, enhanced crop health, time savings, adaptation to changing conditions, and integration with technology. By reducing the need for unnecessary sprays, improving the precision of treatments, and minimizing environmental impact, these models contribute to more sustainable, cost-effective, and efficient agricultural practices. Forecasting plant diseases presents a proactive approach to managing and mitigating the impact of diseases on crops and vegetation.

This Research Topic highlights: 1) latest methods and techniques for developing data-based (empirical) models and process-based (mechanistic) models, and 2) approaches and case-study examples of plant disease models for supporting strategic, tactical, and operational decision-making in crop protection. Aligned with these aims, the topic sought to spur discussion on critical research, development, and technology-transfer (e.g., technology adoption) aspects, namely: Data collection, Timely-control measures, Economic benefits, Integrated crop and pest management (IPM) strategies, Crop diversification, Advances in technologies and tools, Early-warning systems, and Collaboration and communication among farmers, agricultural extension services, researchers in academic/university, corporate, and government institutions.

The Research Topic comprises 19 Original Research articles, and 100 authors from around the globe. Since the launch and Call for Papers, this Research Topic has been viewed 82,589 times with 20,070 article downloads (as of June 30, 2026). Contributed papers present new methodological and technological innovation, findings, and/or insights and are organized and discussed under four themes: (1) Deep Learning and Computer Vision for Plant Disease and Pest Detection, (2) Remote Sensing, Hyperspectral & Multi-Source Approaches in Plant Disease Monitoring, and, (3) Modeling, Forecasting for Crop Diseases and Invasives. These themes were identified with the aid of an artificial-intelligent (AI)-enabled General Pre-trained Transformer (GPT) tool (RADIA-AI) developed by the AI Innovation Hub (Industry, Science and Economic Development (ISED) and Communications Research Council (CRC), Canada).

## Theme 1: deep learning and computer vision for plant disease & pest detection

This theme includes research focusing on the development, optimization, and application of deep learning (CNNs, MLPs, Transformers, YOLO, etc.) for leaf segmentation, detecting crop diseases, and pests. Papers here cover both detection/classification and segmentation using advanced computer vision techniques.

Qiu et al. track movements and behaviors of the litchi pest (*Thalassodes immissaria*), a major pest in litchi and longan fruit tree orchards, in 3D using a binocular vision observation system for nighttime observation and thermal infrared imagery using YOLOX-GMM and SORT-Pest algorithms to reconstruct movement trajectories. The YOLOX, SORT-Pest, and GMM algorithms are widely applied motion target detection algorithms in image processing. YOLOX is a high-performance, open-source object detection model that has revolutionized the YOLO family by transitioning to an anchor-free architecture to simplify the detection process, enabling faster inference. The SORT-Pest tracking algorithm is derived from SORT (Simple, Online, and Real-time Tracking) which is a multiple object tracking framework that uses a Convolutional Neural Network (CNN) detector for identification, a Kalman filter to predict target movement, and the Hungarian algorithm for association. These improvements in the methodology for insect behavior analysis enable better understanding of behavior patterns for developing an effective management strategy for this pest.

Li et al. explore improvements in deep learning in real-world field environments and constraints to provide a practical solution to pest monitoring in rice (e.g., complex backgrounds, limited computational resources). They replace the standard convolutional operations with computationally efficient ghost module in the YOLOv8n’s backbone structure, reducing parameters by 40,458 while maintaining feature richness. A Context Aggregation (CA) module was applied to large and medium-sized feature maps, which enhanced low-level feature representation by fusing global and local context with proved effective for detecting occluded pests. In addition, they apply a Shape-IoU module which improved bounding box regression by accounting for target morphology and SlideLoss which addresses class imbalance by dynamically adjusting sample weighting during training. They validate their model (GhostConv-CA-YOLOv8n) on the Ricepest15 dataset and achieved 90% precision and 82.3% recall, achieving 4.49%, 5.452%, and 3.407% improvement in F1-score, precision, and recall on the IP102 (A Large-Scale Benchmark Dataset for Insect Pest Recognition) benchmark.

Yang et al. present new findings from assessing a novel framework (SRNet-YOLO) for detecting tiny and very tiny pests in cotton fields. They compile a large imagery data set called Cotton-Yellow-Sticky-2023 of pests collected by yellow sticky traps. Their framework includes a YOLO version 8 feature extraction module, a feature map super-resolution reconstruction module, and a fusion mechanism based on BiFormer attention.

Li et al. present a model called YOLO-Leaf that utilizes Dynamic Snake Convolution (DSConv) for robust feature extraction, employs BiFormer to enhance the attention mechanism, and introduces IF-CIoU to improve bounding box regression for increased detection accuracy and generalization ability. This model significantly outperforms existing models in terms of detection accuracy, achieving mAP50 scores of 93.88% and 95.69% (based on Plant Pathology 2020-FGVC7 and Plant Pathology 2021-FGVC8 datasets). The model provides an accurate and effective approach for detecting disease in apple where color information is crucial for identifying disease types. They envision further improvements to better identify disease under field conditions (i.e., complex backgrounds and small disease spots/areas) that can interfere with the identification of disease regions, leading to false positives or missed detections. The authors also aim to further explore multimodal data fusion techniques for combining image data with other types of microsensor data (e.g., temperature, relative humidity) to further improve the accuracy and robustness of disease detection.

Hu et al. construct an imagery database (20,000 images) for four common types of diseases in rice: bacterial leaf blight, rice blast, brown spot, and tungro disease. They introduce the DepMulti-Net model for rice disease and pest identification, which uses VGG16 as the baseline model (with thirteen convolutional layers and three fully connected layers), increasing its model depth using convolutional blocks and the integration of a multi-scale feature fusion module. They design this module to enhance the model’s ability to extract disease features from complex backgrounds. The model has 13.50M parameters and achieved an average accuracy of 98.56% in identifying the four types of rice diseases, outperforming common convolutional neural networks (CNNs): AlexNet, VGG16, ResNet18, DenseNet121, MobileNetV2, and ShuffleNetV2.

Ashurov et al. introduce modifications to the MobileNetV2 architecture (based on its efficient feature extraction capabilities with depth wise separable convolutions) aimed at enhancing its efficacy in plant disease classification tasks. They integrate the Squeeze-and-Excitation (SE) module and incorporate residual skip connections to refine feature representation and improve the robustness of their model. An additional, fully connected layer with L2 norm regularization is included to enhance the efficacy of the model and prevent overfitting. The model outperforms five state-of-the-art models (i.e., VGG16, NasNetMobile, ResNet50, Inception, Xception) for validation metrics of Accuracy, Precision, Recall, F1 Score, inference time, and model size. They aim to further address data imbalance by employing sophisticated data augmentation techniques, and address interpretability of the model and its decision-making processes, especially when adapted and applied to a wider range of plant diseases and species. They also seek to incorporate predictive analytics for forecasting disease outbreaks to improve the model’s proactive capabilities, allowing producers to take preventative measures.

Lu et al. explore the detection of disease in yam, an important medicinal and edible crop. To address current challenges such as leaf overlapping, uneven lighting and irregular disease spots in complex environments, they propose a model (BiSeNeXt) to improve disease segmentation accuracy. This model includes a dynamic feature extraction block (DFEB) for more precision in identifying leaf and disease edge pixels. They also integrate dynamic receptive-field convolution (DRFConv) and pixel shuffle (PixelShuffle) downsampling for reducing lesion omission in the detection process. Asymmetric multi-scale attention (EAMA) provides asymmetric convolution for lesion identification, and a PointRefine decoder adaptively selects uncertain points in the image predictions and refines them point-by-point enabling more accurate segmentation of leaves and spots. Their model achieved a 97.04% intersection over union (IoU or Jaccard Index) for leaf segmentation and an 84.75% IoU for disease segmentation, where IoU is the ratio of the overlapping area of a predicted box/mask and the actual ground truth.

Li et al. propose a method for overcoming the problem of temporal and spatial irregularity of plant diseases, which may result in insufficient image data for certain diseases, challenging traditional deep learning methods that rely on large amounts of manually annotated data for training. Specifically, they develop a method based on the Feature Adaptation Score (FAS) metric, which calculates the FAS for each feature layer in the Swin-TransformerV2 structure. They identify a network structure suitable for few-shot plant disease classification without training the network, by leveraging the strict positive correlation between FAS scores and test accuracy. Using this network structure, they develop a Plant Disease Feature Calibration (PDFC) algorithm, which uses extracted features from the PlantVillage dataset to calibrate features from other datasets. This approach significantly improves the accuracy of few-shot plant disease classification, surpassing existing state-of-the-art models.

Li et al. address the current limitations of spot segmentation models for rice diseases, which have high computational overhead and are difficult to deploy in field environments. They develop a lightweight rice leaf spot segmentation model for rice blast, brown spot and bacterial leaf blight. The lightweight feature extraction network MobileNetV3_Large (MV3L) was adopted as the backbone of the model. They develop a multi-scale detail enhancement (MSDE) module to improve decision-making ability of the model in transitional regions such as spot gaps, and to improve the sticking and blurring problems at the boundary of spot segmentation. They also propose a PagFm-Ghostconv Feature Fusion (PGFF) module to significantly reduce the computational overhead of the model. Furthermore, they incorporate a coordinate attention (CA) mechanism before the PGFF module to improve model robustness in complex environments. They use a hybrid loss function integrating Focal Loss and Dice Loss to mitigate class imbalance between disease and background pixels in imagery. The MMPC-DeepLabv3+ model obtains high performance over DeepLabv3+, U-Net, PSPNet, HRNetV2, and SegFormer, achieving an optimal balance between recognition accuracy and computational efficiency.

Zhang et al. present a novel model (EDGE-MSE-YOLOv11) for detecting rice diseases. This model integrates a unified Tri-Module Lightweight Perception Mechanism (TMLPM) with three components: multi-scale feature fusion (C3K2 MSEIE), attention-guided feature refinement (SimAM), and efficient spatial down-sampling (ADown). These components significantly enhance the model’s ability to detect multi-scale and small disease targets under complex field conditions. The EDGE-MSE-YOLOv11 model, benchmarked against the YOLOv11n model and using an augmented disease imagery dataset comprising 11,617 images, originally collected from experimental paddy fields located in Jiangsu Province, China in 2022–2023), with a training set contains 8,130 images, validation set of 2,324 images, and independent testing set of 1,163 images. The model improves detection precision (from 85.6% to 89.2%), recall (from 82.6% to 86.4%), mAP@0.5 (from 90.2% to 92.6%), and mAP@0.5:0.95 (from 63.7% to 70.3%). Their future work aims to explore cross-domain generalization and deployment optimization using lightweight techniques such as quantization, pruning, and transformer-based enhancements to increase the model robustness and scalability in serving as a disease diagnosis system for smart agriculture.

Abulizi et al. propose an improved tomato leaf disease detection method, DM-YOLO, based on the YOLOv9 algorithm. This model uses dynamic up-sampling within the feature fusion backbone network to enhance its ability to extract features of small lesions and suppress the interference from the background environment. It also uses the MPDIoU loss function (see references therein) to enhance the learning of the details of overlapping lesion margins to improve the accuracy of localizing overlapping lesion margins. This is because tomato leaf disease symptoms differ in shape and size, with lesion locations overlapping to variable extents. This makes it challenges to localize lesions and extract detailed features of their margins. The model’s precision (P) was 92.5%, evaluated using a tomato leaf disease dataset. The original dataset consists of images taken in both the outdoor and laboratory environment but was expanded to 4124 images using augmentation methods such as shift, random cropping, rotation, scaling, and brightness control to increase the diversity and richness of the training sample images.

Fang et al. address the current challenges in pepper leaf segmentation for the monitoring of pepper leaf diseases, which include computational inefficiency, excessive parameterization, and inadequate utilization of edge information. An Adaptive Multi-Scale MLP (AMS-MLP) framework was then introduced, which integrates a Multi-Path Aggregation Module (MPAM) and a Multi-Scale Context Relation Mask Module (MCRD) to refine object boundaries in pepper leaf segmentation. The proposed method was validated through extensive experiments on three pepper leaf datasets with varying backgrounds. The results demonstrate mean Intersection over Union scores of 97.4%, 96.9%, and 97.9%, and F1 scores of 98.3%, 97.9%, and 98.51% across the datasets, respectively. The proposed method then significantly improves the accuracy and efficiency of pepper leaf image segmentation.

## Theme 2: remote sensing, hyperspectral & multi-source approaches in plant disease monitoring

This theme includes studies that involve hyperspectral imaging, UAV sensing, remote sensing, and other data-intensive or sensor fusion approaches for plant disease monitoring, pest tracking, and landscape-level ecosystem health surveillance. It also covers spatial/temporal modelling for large-scale disease and pest outbreaks.

Cross et al. acquire leaf-level and canopy-level spectra from hyperspectral imaging technology (hereafter, HSI) and assessed random forest models to assess the ability of machine learning to accurately classify wheat stripe rust (WSR) severity. They infer that severity classes have distinct spectral features that become more pronounced as the disease symptoms become more severe, whereby a canopy-level model with 5 severity classes, achieved an overall accuracy of 78%, increasing to 96% using an “off-by-one” assessment (i.e., a classification correct if it falls within one class of the expert human label). They also identify that the addition of a single narrowband observation around 640 nm has the potential to significantly improve early-stage wheat rust detection. They demonstrate that machine learning in combination with a few carefully selected hyperspectral reflectance wavebands can precisely remotely monitor and manage wheat stripe rust to limit crop damage. Their approach utilizes backward feature elimination that reduces the entire reflectance spectrum by removing the least significant 10% of features, to leave a set of wavebands that are most important for prediction.

Zhang et al. utilize HSI to investigate early detection of pear anthracnose through spectral features, vegetation indices (VIs), and texture features (TFs). Several competing machine learning models for classifying images were assessed, namely: Support Vector Machine (SVM), Extreme Learning Machine (ELM), and Back Propagation Neural Network (BPNN). Their results demonstrate that models constructed using a fusion of multi-source features outperform those using single features, achieving the highest accuracy of 98.61% in disease detection.

Retkute and Gilligan explore Banana Bunchy Top Virus (BBTV) disease control and prediction, presenting a novel methodology for deriving probabilistic production maps for Tanzania and Ogun State in Nigeria based on high-resolution remote sensing information (i.e., canopy height and net- difference vegetation index, NDVI). In addition, they parameterize a spatially explicit susceptible-infection epidemiological model from available disease survey data. This model tracks the infection status at the 1 km resolution. The data and code to produce a banana production map that is available in a GitHub repository. Results emphasize the importance of having data on the presence and absence of Banana Bunchy Top Virus (BBTV) at different stages of epidemics for disease control and prediction.

Zhang et al. successfully improve tea pest and disease detection in imagery with the occlusion of multiple targets at once with complex backgrounds. A wavelet transform/convolution increases detection efficiency, and multiscale, multi-head self-attention improved with small target detection. A context-guided spatial feature pyramid network was also integrated that helped to achieve significant improvements in the robustness and accuracy of target detection in complex scenes. They deploy their methodology (WMC-RTDETR) on the Raspberry Pi platform serving as a low-cost tool for real-time monitoring of pest and disease in tea plantations, and in the future may be assessed for other agricultural crops.

## Theme 3: modeling and forecasting of crop diseases and invasives

This theme includes research primarily focused on predictive modeling of disease or pest population dynamics, epidemics, or future spread. It also includes system-level approaches, early warning, risk assessment, and management implications for agricultural sustainability and food security.

Kalisetti et al. develop a forecasting system for fall armyworm (*Spodoptera frugiperda*) in corn (maize), testing several different models: GARCH with exogenous variables (INGARCHX), Support Vector Regression with climate inputs (SVRX), and Artificial Neural Network with climate inputs (ANNX). These models integrate climate variables such as air temperature, rainfall, and relative humidity. During the training phase, the ANNX model delivered the best performance compared to both SVRX and INGARCHX. During testing, the ANNX model consistently outperforms the alternatives. Model validation testing shows that the ANNX model can predict fall armyworm population surges with 80% accuracy, one week ahead, providing sufficient early-warning for enabling pest control actions to be taken.

Fan et al. assess the invasion potential of *Bidens pilosa* L., a globally invasive weed originating in tropical America, under current and future climates. This weed infests thirty-one economically vital crops in over forty countries. They fit the MaxEnt species distribution model (i.e., for presence-only data) (see Elith et al., 2011) to available occurrence data assimilated for this weed around the world. The MaxEnt model is a machine-learning algorithm and software for predicting habitat suitability. It identifies the environmental conditions (like climate and elevation) where a species is most likely to survive and creates a map of suitable habitats. It is particularly useful when you only have “presence-only” data (locations where a species has been seen). Key factors influencing the weed spread, including temperature, precipitation, and human influence, were identified. Under current climate conditions, coastal regions have the highest invasion potential, with suitable areas decreasing with distance from the sea. Their findings suggest a likely decline of suitable habitats in tropical regions and an expansion into temperate regions, with climate suitability decreasing under higher temperatures. Additionally, historical reconstructions emphasize that the rapid spread of the species was facilitated by maritime trade routes. They identify significant distributions in southeastern Asia, eastern Oceania, eastern Africa, southern North America, and southeastern South America. Under future scenarios, the modeling predicts this weed’s distribution to decline, but in colder regions, the model predicts its distribution will expand. They propose management strategies that emphasize the need for enhanced control measures in high-risk areas and conservation efforts in their native range.

Li et al. assess the establishment risk, the local dispersal patterns, risk areas, and economic losses of the bacterial pathogen that causes fire blight (*E. amylovora*) that poses a serious threat to pear and apple production worldwide. They integrate the MigClim species distribution model with a minimum cost arborescence approach (Engler and Guisan, 2009), and conduct Monte Carlo stochastic simulations. MigClim is an R Statistical Language package that adds dispersal constraints to the projections generated by models like MaxEnt. MaxEnt simply maps where a species *could* live in the future, whereas MigClim simulates how fast the species can *move or spread* to reach those new habitats over time, accounting for natural barriers, seed dispersal limits, and landscape fragmentation. Their findings indicate that its global distribution and host overlapping areas (i.e., pear and apple) are expected to expand under future climatic conditions.

## Future outlook

This Research Topic collection showcases recent methodological advances in the prediction of disease and pest risk, early detection, spread, and distribution patterns under climate change. The studies focus on diseases and pests that are major threats to agricultural crop production and global food security and include a broad range of methods - from widely applied statistical, machine-learning, and deep learning approaches to latest methodological advancements and hybrid methods.

In this Research Topic, most contributions focused on machine-learning and deep learning in computer vision of pests and diseases. Theme 1 studies involve the construction of training databases of RGB/multispectral/hyperspectral imagery data and the use of pest trap data, presence-absence survey data, and microsensor data. The studies address current challenges involving the detection of small disease areas/spots, tiny and occluded pests. and complex backgrounds. Innovation using asymmetric convolution to identify lesions on leaves, hybrid methods and improved loss functions that increase detection/recognition accuracy and computational efficiency. The authors highlight future work aimed at increasing the diversity and richness of training datasets for detecting multiple diseases simultaneously, cross-disciplinary applications, the use of multi-modal data fusion (MMDF) i.e., combining disparate data streams of different dimensionality, resolution, and type, including increased use of microsensor data (e.g, ambient temperature and relative humidity) as input of forecasting models to predict outbreaks.

Theme 2 studies demonstrated the classification of disease severity (wheat stripe rust, WSR) using hyperspectral data, identifying that a narrowband observation (640 nm) has the potential for improving early-stage detection for WSR, along with the use of band selection and importance weighting to improve prediction. Fusion models combining vegetation and texture features were found to outperform single feature models in the case of pear anthracnose detection. Similarly, combining remote-sensing indices with disease survey data improved the parameterization and application of an epidemiological regional-scale model for Banana Bunchy Top Virus (BBTV).

Theme 3 studies demonstrated forecasting of fall armyworm (*Spodoptera frugiperda*) in corn that used climate information and generates early-warning forecasts one-week ahead with 80% accuracy. The MaxEnt species distribution model is applied to the invasive weed *Bidens pilosa* L. and insights on its climate suitability and global spread are identified: declining in high temperature regions, with expansion into colder regions. The MigClim species distribution model, applied to the bacterial pathogen that causes fire blight (*E. amylovora*) on pear and apple, indicates that the distribution of this disease is expected to expand under future climatic conditions.

While the studies in this Frontiers Research Topic demonstrated significant advancements in addressing many current research domain challenges, the studies point to several domains of emerging challenges where future research is critically needed. These domains are: 1) model generalization across regions, 2) interoperability of operational forecasting systems, and, 3) practical adoption by growers. Yan et al. (2026) recently conducted a broad and systematic evaluation of existing research in crop disease detection, prediction, and early-warning from the dimensions of sensors and systems, methods and algorithms, and applications. Their review also identified similar challenges in developing effective and reliable practical applications for crop disease and pest monitoring, prediction, and management, in part, due to imprecise detection under natural environmental changes, crop canopy occlusion, ambiguous disease characteristics, and the lack of fusion or integration of multiple sensors and technologies.

As highlighted by Giakoumoglou et al. (2026) in another recent review of AI in plant disease and pest detection, there are three representative scales of interest in plant pest and disease research; *population-level* pest monitoring, *field-scale* surveillance, and *plant-centric* detection. In addressing the emerging challenges 1–3 mentioned above, it is important to also consider these three relevant scales of interest. This is because each of these modeling scales has different acquisition and data requirements for training. Also, each scale has different validation requirements for upscaling and extrapolating across different regions necessary to be generalizable whereby their predictions to be sufficiently robust and accurate. In addition, each scale is most relevant to the needs and interests of different stakeholders, such as policy-makers, extension specialists, and producers (farmers, growers). Addressing these emerging challenges will require research on the integration of best-performing models across the three scales and/or across different model architectures so that operational systems can become more interoperable and flexible and, in turn, more transferable and widely applied. The needs of different stakeholder groups also need to be taken into account, alongside developing cooperative strategies to build trust and understanding of model methodologies and associated operational tools. One must not lose sight that the shared long-term challenge is to achieve real-world impact, whereby stakeholders value and use these new, powerful tools to guide improvements in their agronomic management practices and/or optimize their decision-making to reduce pest and disease pressure and future risks.
